# A systematic review of dietary changes after migration in women: a focus on schizophrenia

**DOI:** 10.1192/j.eurpsy.2025.1464

**Published:** 2025-08-26

**Authors:** M. E. Chavez, M. Natividad, B. Palacios-Hernández, A. Balagué, E. Rial, J. Paolini, R. Penades, J. Cobo, M. Salvador, A. Vallet, R. León, E. Izquierdo, J. A. Monreal, A. González-Rodríguez

**Affiliations:** 1 Department of Mental Health, Mutua Terrassa University Hospital. University of Barcelona, Terrassa, Spain; 2 Perinatal Mental Health Research Laboratory CITPsi-UAEM, Autonomous University of the State of Morelos, Cuernavaca, Mexico; 3 Barcelona Clinic Schizophrenia Unit (BCSU)., Institute Clinic of Neurosciences. Hospital Clinic of Barcelona. University of Barcelona. IDIBAPS, CIBERSAM, Barcelona; 4 Department of Mental Health, Parc Tauli University Hospital. Autonomous University of Barcelona (UAB). I3PT, CIBERSAM, Sabadell; 5 Department of Mental Health, Mutua Terrassa University Hospital. University of Barcelona. CIBERSAM, Terrassa, Spain

## Abstract

**Introduction:**

The impact of migration on cardiovascular risk factors have been reported to be gender-specific. Obesity and cardiovascular disease are increased in those who migrate to Western countries.

**Objectives:**

Our aim is to investigate changes in the dietary habits of women after migration, especially in schizophrenia women.

**Methods:**

A systematic review was performed in PubMed, Scopus and PsycINFO databases from inception to October 2024 according to the PRISMA statement. Search terms: (diet OR food OR “dietary acculturation”) AND migration AND women. Studies were included if they were focused on dietary changes after migration in women. In a second step, we conducted electronic searches to find additional papers on schizophrenia.

**Results:**

A total of 2046 records were screened, of which 36 studies were included.

(1) Socio-clinical scenarios of migration: a)Latin-American (n=5), b)African (n=7), c)Asian (n=17), Europe (n=2). Results: Weight gain after migration to developed countries, reduced dietary diversity and limited access to culturally appropriate foods are common (poor traditional-food trajectories). Early stages of migration are critical. Model of dietary transition during pregnancy (3 stages) and risk of gestational diabetes.

(2) Transnational migration (rural-urban, n=5). Indian women had higher intakes of both fruit and vegetables and fat. Migration from rural-to-urban and urban-to-urban areas was associated with obesity risk. Exception: rural migrants to Mongolia’s capital maintaining traditional lifestyles. Few studies focus on women with schizophrenia.

**Conclusions:**

The dietary habits of migrant women may have implications for future chronic disease risk, particularly for those with schizophrenia. Early culturally sensitive weight-loss interventions for migrant women are recommended.

**Key-words:**

Migration; Diet; Dietary acculturation; Schizophrenia; Women.

**Disclosure of Interest:**

None Declared

